# 基于影像学诊断非小细胞肺癌肿大淋巴结良恶性的研究进展

**DOI:** 10.3779/j.issn.1009-3419.2023.101.01

**Published:** 2023-01-20

**Authors:** Kai QIN, Xiaolong FU

**Affiliations:** 200030 上海，上海交通大学医学院附属胸科医院放疗科; Department of Radiotherapy, Shanghai Chest Hospital, School of Medicine, Shanghai Jiao Tong University, Shanghai 200030, China

**Keywords:** 肺肿瘤, 影像学, 淋巴结, 影像组学, 深度学习, Lung neoplasms, Radiography, Lymph node, Radiomics, Deep learning

## Abstract

非小细胞肺癌（non-small cell lung cancer, NSCLC）在影像学上可见肿大淋巴结，但其良恶性难以直接判断，为肿瘤分期及设计放疗靶区带来了困难。临床上常依据淋巴结短径≥1 cm或最大标准摄取值≥2.5诊断淋巴结为恶性，但这些诊断标准敏感性和特异性均较低，难以满足临床需要。近年来，基于其他影像学参数诊断淋巴结良恶性取得了众多进展，同时随着影像组学、深度学习等技术的发展，通过挖掘肿大淋巴结区域的影像信息建立模型进而提升淋巴结良恶性诊断准确性展现出巨大的应用前景。本文旨在综述近年来基于影像学诊断NSCLC肿大淋巴结良恶性的研究进展，以便临床工作中更准确且无创地评估淋巴结状态。

## Abstract (en)

Non-small cell lung cancer (NSCLC) can be detected with enlarged lymph nodes on imaging, but their benignity and malignancy are difficult to determine directly, making it difficult to stage the tumor and design radiotherapy target volumes. The clinical diagnosis of malignant lymph nodes is often based on the short diameter of lymph nodes ≥1 cm or the maximum standard uptake value ≥2.5, but the sensitivity and specificity of these criteria are too low to meet the clinical needs. In recent years, many advances have been made in diagnosing benign and malignant lymph nodes using other imaging parameters, and with the development of radiomics, deep learning and other technologies, models of mining the image information of enlarged lymph node regions further improve the diagnostic accuracy. The purpose of this paper is to review recent advances in imaging-based diagnosis of benign and malignant enlarged lymph nodes in NSCLC for more accurate and noninvasive assessment of lymph node status in clinical practice.

肺癌发生约占癌症总数的11.6%，位居第二；肺癌死亡约占癌症相关死亡的18.4%，位居首位；其中非小细胞肺癌（non-small cell lung cancer, NSCLC）约占肺癌的85%^[1,2]^。N分期是NSCLC肿瘤分期的重要部分，是选择治疗手段和判断预后的重要依据。近年来，尽管影像学技术进步巨大，但其影像学评估准确性仍欠佳^[3,4]^，众多其他影像学参数也表现出对淋巴结的诊断价值。通过影像组学、深度学习等新兴技术挖掘淋巴结区域影像中蕴含的丰富特征进而诊断淋巴结良恶性也展现出广大的应用前景。本文旨在综述近年来基于影像学诊断NSCLC患者肿大淋巴结良恶性的研究进展，以便临床医生更准确而无创地评估NSCLC患者的淋巴结状态。

## 1 基于影像学诊断肿大淋巴结良恶性的临床意义

准确诊断NSCLC肿大淋巴结的良恶性是明确N分期的重要环节，影响着后续治疗手段的选择和预后判断等^[5]^。临床上常依据电子计算机断层扫描（computed tomography, CT）淋巴结短径≥1 cm或正电子发射计算机断层成像（positron emission tomography/CT, PET/CT）上最大标准摄取值（maximum of standardized uptake value, SUVmax）≥2.5诊断淋巴结为恶性。但由于淋巴结反应性增生、肺部炎症、肉芽肿性疾病等良性病变也可引起淋巴结增大及代谢增高，这些标准的特异性较低^[6]^。由于目前影像学诊断标准的准确率较低，对于影像学上的肿大淋巴结，美国国立综合癌症网络（National Comprehensive Cancer Network, NCCN）等指南建议进一步行有创性检查如超声内镜引导下的经支气管针吸活检、纵隔镜等获取淋巴结组织样本以明确淋巴结良恶性，以便更准确地进行N分期^[7]^。然而有创性检查存在造成创伤、患者接受程度低、部分淋巴结由于解剖学位置难取样造成敏感性降低等问题，此外对于拟接受放疗的患者，往往存在多站、多枚淋巴结肿大，每个淋巴结取活检不可行等问题，因而有创性检查的临床使用常受到限制^[8]^。对于以放疗作为主要治疗手段的患者，淋巴结放疗总体原则是对于明确存在转移的淋巴结做局部放疗，而不实施淋巴引流区域预防性照射，这就要求对每个肿大淋巴结是否存在转移作出精准判断^[9]^。基于目前影像学诊断淋巴结良恶性的标准，在临床放疗实践中，不可避免地存在漏照或过度照射等问题。对每个肿大淋巴结进行有创的病理学检查，患者依从性和可操作性较差，这意味着基于影像学更准确地诊断肿大淋巴结良恶性对优化治疗靶区有重要意义。

## 2 基于CT诊断肿大淋巴结良恶性

CT是NSCLC患者的基本检查之一，对临床评估患者是否存在淋巴结转移及转移部位具有重要价值。目前临床上主要将CT上淋巴结短轴直径≥1 cm作为恶性淋巴结诊断标准，但其诊断准确性欠佳，近年来发现其他CT参数如淋巴结CT值、淋巴结形状等在良恶性淋巴结之间存在差异，可辅助诊断淋巴结良恶性。

### 2.1 淋巴结短轴直径

基于CT上淋巴结短轴直径判断淋巴结良恶性是目前最广泛使用的诊断标准，淋巴结短轴直径≥1 cm被认为是恶性的，<1 cm则考虑为良性。然而炎症、肉芽肿、淋巴结反应性增生等良性病变同样可以引起淋巴结短径≥1 cm，并且肿瘤转移灶较小时淋巴结可保持正常大小，其识别恶性淋巴结的敏感性和特异性分别约为55%和81%^[10]^，阳性预测值和阴性预测值分别为56%和83%^[11]^。尽管根据淋巴结短轴直径判断恶性淋巴结的敏感性和特异性低，但淋巴结短轴长度越大则其为恶性的可能性越大，Prenzel等^[12]^报告NSCLC患者中短径<5 mm、5 mm-9 mm、10 mm-14 mm、≥15 mm的淋巴结分别有4.0%、10.7%、25.3%、40.1%的淋巴结为恶性。

### 2.2 淋巴结CT值

淋巴结的CT值反映了其密度，可辅助诊断淋巴结的良恶性。CT值>70 HU的淋巴结常表明其为良性，有研究^[13,14]^认为淋巴结具有高CT值通常意味着其为肉芽肿性炎症（如肺结核）或淋巴结钙化等。Lee等^[15]^分析了271个淋巴结的CT值直方图，发现71.0%的恶性淋巴结CT值中位值在25 HU-45 HU，而78.9%的良性淋巴结CT值<25 HU或>45 HU；CT值为25 HU-45 HU的淋巴结中58.2%为恶性，CT值<25 HU或>45 HU的淋巴结仅16.7%为恶性。Flechsig等^[16]^报告恶性淋巴结CT密度中位值显著高于良性淋巴结，CT值>20 HU的淋巴结中77%为恶性；CT值<20 HU的淋巴结中18%为恶性，他们认为恶性淋巴结CT值较高可能是由于淋巴结脂肪门结构丧失引起，而淋巴结脂肪门的存在通常意味着淋巴结为良性。邵亭亭等^[17]^利用淋巴结与纵隔血池密度的比值诊断淋巴结良恶性，其曲线下面积（area under curve, AUC）为0.755，密度比≤0.9记为恶性淋巴结，敏感性为68.9%，特异性为79.9%。

### 2.3 淋巴结形状

Yin等^[18]^发现使用淋巴结长轴长度/短轴长度之比（L/S），较使用淋巴结短轴长度能更好地判断淋巴结良恶性。他们发现大多数情况下，正常淋巴结为椭圆形，而当淋巴结被恶性肿瘤侵犯时，淋巴结的形状趋于圆形，失去了正常的椭圆形。但对于反应性淋巴增生，长短径的增加往往遵循一定的比例，维持正常的椭圆形。他们报道恶性淋巴结L/S平均为1.60，良性淋巴结L/S平均为2.03；86.48%的转移淋巴结的L/S<2，而78.57%的良性淋巴结的L/S≥1.5，但计算淋巴结L/S需先得到淋巴结立体图像，相比测量淋巴结短轴更加复杂。

通过CT诊断淋巴结良恶性主要基于肿瘤细胞可引起淋巴结结构改变，包括淋巴结的大小、形状、密度等。但对于微小转移灶，CT观测到的淋巴结结构可能保持正常，同时这些结构改变在炎症、肉芽肿、淋巴结反应性增生等良性病变中也可出现，因此其敏感性和特异性均低。相较于临床常用的平扫和增强CT，近些年发展的能谱CT也表现出较高的诊断价值，朱巧等^[19]^报道定量能谱参数如静脉期标准化碘浓度可通过反映淋巴结内部血供情况进而鉴别淋巴结良恶性，其准确性为77.9%，优于通过淋巴结短径诊断，具有潜在应用价值。

## 3 基于PET/CT诊断肿大淋巴结良恶性

PET/CT是一种可有机融合PET代谢图像和CT解剖图像的影像学技术，其中PET常基于示踪剂^18^F-氟脱氧葡萄糖（^18^F-fluorodeoxyglucose, ^18^F-FDG）在肿瘤细胞中摄取更高进行成像。PET/CT诊断NSCLC淋巴结良恶性主要依据淋巴结的SUVmax，一般认为SUVmax≥2.5的淋巴结为恶性。PET/CT根据淋巴结代谢增高，可发现正常大小淋巴结中的转移灶，因而其诊断恶性淋巴结的敏感性显著高于CT。然而炎症、结核病等非肿瘤转移因素同样可引起淋巴结代谢增高，使得其诊断特异性降低^[20]^。此外受限于代谢成像分辨率有限，微小的高代谢灶由于部分容积效应无法在PET/CT上得到体现，仍然存在假阴性的问题^[21]^。PET/CT诊断性能受淋巴结大小影响，对短径≥1 cm的淋巴结，PET/CT敏感性显著高于短径<1 cm的淋巴结（74% vs 40%），但特异性显著降低（81% vs 98%），同时准确性也显著降低（78% vs 90%）^[22]^。此外SUVmax测量的准确性受到一些干扰因素的影响，如血糖水平、呼吸运动、示踪剂的摄取时间等^[23]^。临床常用的淋巴结SUVmax仅代表特定时间特定区域内具有最高体素值的单一代谢焦点，近年来其他代谢参数也表现出对淋巴结良恶性独特的诊断价值。

### 3.1 淋巴结SUVmax

恶性淋巴结由于存在肿瘤细胞，代谢变得更加活跃，可摄取更多的^18^F-FDG，在PET/CT上具有更高的SUVmax，但炎症等因素同样会使得淋巴结代谢增高进而导致假阳性，而转移灶较小时则可产生假阴性诊断。关于淋巴结SUVmax的最佳截止值尚有争论，目前一般采用SUVmax≥2.5作为恶性淋巴结的诊断标准。一项荟萃分析^[24]^报告以淋巴结SUVmax≥2.5为标准，PET/CT诊断恶性淋巴结的汇总敏感性和特异性分别为81.3%和79.4%，而以淋巴结活动大于本底背景为标准的敏感性和特异性分别为77.4%和90.1%。Lee等^[15]^发现使用4.0作为SUVmax的截止值，显示出对恶性淋巴结最高的诊断能力，其特异性较高为94.5%，而敏感性稍低，为70.4%。Kumar等^[25]^发现适当增加SUVmax截止值可提高特异性，同时保持可接受的敏感性，他们发现当2.5作为截止值时，敏感性为93%，特异性为40%，而当6.2作为截止值时，敏感性为87%，特异性为70%。不同研究间SUVmax最佳截止值存在差异，可能与纳入的研究人群及测量条件有关。

### 3.2 淋巴结体积代谢参数

SUVmax仅代表特定区域内具有最高体素值的单一代谢焦点，基于体积的代谢参数如代谢肿瘤体积（metabolic tumor volume, MTV）、总病变糖酵解（total lesion glycolysis, TLG）在诊断良恶性淋巴结方面的价值也得到了关注。Nakanishi等^[26]^发现恶性淋巴结的SUVmax、MTV、TLG3.5以及淋巴结与原发肿瘤SUVmax的比率（lymph node-to-primary tumor ratio of SUVmax, LPR）均显著高于良性淋巴结。多变量分析提示淋巴结的LPR可替代SUVmax作为诊断淋巴结良恶性的独立预测因素。此外，亚组分析提示在肺鳞癌中LPR预测效果最佳，而在肺腺癌中TLG3.5预测效果最佳。

### 3.3 淋巴结双时间点（dual-time-point, DTP）成像参数

DTP ^18^F-FDG PET/CT在区分良性和恶性病变方面具有优势，其基本原理是肿瘤中^18^F-FDG的摄取可能会随着时间的推移而增加，而在正常和非恶性病变中^18^F-FDG的摄取随时间的推移可能会保持稳定或减少^[27]^。相较于某时刻测得的SUVmax，^18^F-FDG摄取随时间的变化可能是一个更好的区分良恶性疾病的代谢参数。Lee等^[28]^报道DTP PET/CT对诊断NSCLC纵隔淋巴结具有良好性能，早期图像显示灵敏度为0.80，特异性为0.83，延迟图像显示灵敏度为0.84，特异性为0.87。基于早期和延迟图像计算的保留指数或SUVmax的百分比变化在诊断纵隔淋巴结方面优于单独早期或延迟的DTP PET/CT图像。

目前常用的示踪剂^18^F-FDG对恶性淋巴结诊断具有较高的敏感性，但由于^18^F-FDG摄取增加在增生、炎症、感染和肉芽肿性疾病中均可存在，因而其特异性较低^[20]^，对恶性淋巴结诊断更敏感和特异的示踪剂仍在探索中。Zhou等^[29]^发现示踪剂^68^Ga-DOTA-FAPI-04在良性淋巴结中摄取显著低于恶性淋巴结，可明显降低淋巴结诊断的假阳性率，然而与^18^F-FDG相比，使用^68^Ga-DOTA-FAPI-04产生了更多的假阴性诊断，他们认为可能是由于小转移灶中成纤维细胞激活蛋白表达较低所致，其诊断性能仍待进一步验证。

## 4 基于磁共振成像（magnetic resonance imaging, MRI）诊断肿大淋巴结良恶性

MRI由于扫描时间较长且肺部运动幅度较大、空气充盈等原因难以对肺部清晰成像，未常规应用于NSCLC胸部检查^[30]^。随着MRI快速成像技术的发展，近几年已有较多研究报道了MRI对诊断恶性淋巴结具有较高的特异性和准确性，但目前尚未广泛应用于临床。MRI中的扩散加权成像（diffusion weighted imaging, DWI）序列可反映组织中水分子的扩散情况，对诊断淋巴结良恶性具有较好的效果，在DWI序列中测量表观弥散系数（apparent diffusion coefficient, ADC）可用于鉴别淋巴结良恶性^[31]^。

ADC值反映了DWI序列中不同方向的分子扩散运动的速度和范围，对鉴别淋巴结良恶性有潜在应用价值。其原理基于恶性淋巴结存在肿瘤细胞，而肿瘤细胞内水分子活动较正常细胞受限，因而其ADC值减低^[32]^。ADC值的常用类型包括平均ADC（mean ADC value, ADCmean）和最小ADC（minimum ADC value, ADCmin），蔡荣芳等^[33]^使用1.88×10^-3 ^mm^2^/s作为ADCmean阈值时诊断恶性淋巴结的敏感度和特异度分别为96.6%和90.3%，而使用1.57×10^-3^ mm^2^/s作为ADCmin阈值时敏感度和特异度分别为98.3%和90.3%。一项荟萃分析^[34]^报道基于DWI序列诊断淋巴结良恶性的汇总敏感性和特异性分别为0.70和0.97，PET/CT的相应值分别为0.69和0.93，这表明PET/CT和MRI的DWI序列均有利于诊断纵隔淋巴结良恶性，诊断能力无统计学差异。

对于诊断淋巴结良恶性，除了目前研究较多的DWI序列，其他序列如短时间反转恢复序列等也被认为存在诊断价值^[35]^。相较于在临床广泛使用的CT和PET/CT，将肺部MRI纳入常规临床实践仍有许多问题待解决，包括进一步验证基于图像的测量方法、建立正常值、证明成本效益和可改善患者预后等^[36]^。

## 5 基于影像组学诊断肿大淋巴结良恶性

影像组学于2012年由Lambin等^[37]^提出，如今已成为一门处理医学影像的新兴学科，影像组学通过自动算法将医学影像中的感兴趣区域（region of interest, ROI）转化为高通量定量特征信息，从而将图像转化为可挖掘的数据。影像组学可帮助医生量化ROI中诸如纹理、形状、小波等难以人工观测的特征，这些特征可通过机器学习算法（如支持向量机、随机森林等）进行挖掘，进而建立数学模型。影像组学特征在反映肿瘤的生物学信息，例如细胞形态、分子和基因表达等方面已取得大量研究成果，可比临床医生更准确地判断病理类型、预后和治疗反应等^[38]^。近年来，基于影像组学诊断肿大淋巴结良恶性的研究已取得大量成果，有望在现有成像技术下进一步提升淋巴结诊断的准确性。

恶性淋巴结由于肿瘤细胞的存在，可有淋巴结结构的改变，如前所述可在大小、密度、形状等方面不同于良性淋巴结。除了可人工观测的特征外，恶性淋巴结还有许多人工难以观测的特征不同于良性淋巴结，例如异质性等，而通过影像组学的特征提取算法可反映这些差异。Andersen等^[39]^分析了29例NSCLC患者的46个纵隔淋巴结，通过对淋巴结区域的增强CT进行纹理分析，他们发现良性和恶性淋巴结的纹理特征存在显著差异，并通过筛选的纹理特征建立逻辑回归模型，其诊断淋巴结良恶性的AUC为0.834。同年Bayanati等^[40]^也发现平扫CT上纵隔淋巴结的纹理特征以及3个表征淋巴结圆形度的形状参数可建立模型区分良性和恶性淋巴结，其AUC为0.87。Sha等^[41]^提取了平扫期、动脉期、静脉期图像的淋巴结影像组学特征用于鉴别淋巴结良恶性，结果显示不同CT相位的影像组学模型都有较高的准确性，其中平扫期模型AUC最高，联合动脉期特征后，其敏感性和阴性预测值可进一步提高。

随着PET/CT应用的增多，基于PET/CT进行影像组学建模也显示出较单纯PET/CT参数有更好的诊断性能。Yoo等^[42]^分析了1,329个纵隔淋巴结的PET/CT影像，提取并筛选影像组学特征，通过增强决策树建立的纵隔淋巴结诊断模型效果最佳，并且在模型结合患者吸烟史和肺部病史后，其准确率和AUC显著高于医生，尤其是对SUVmax偏低的纵隔淋巴结。Xie等^[43]^回顾性分析了263个淋巴结，提取了其在PET/CT中对应区域的CT影像组学特征，通过特征筛选后，纳入14个影像组学特征建立了诊断淋巴结良恶性的淋巴结影像组学评分，其AUC在训练组和测试组中分别为0.849和0.828。此外多变量逻辑回归提示，淋巴结影像组学评分和SUVmax是诊断淋巴结良恶性的独立风险因素，可建立诊断列线图，其在测试组的AUC为0.872，显著高于仅使用淋巴结SUVmax的AUC（0.828）和仅使用淋巴结短轴的AUC（0.729）。Ouyang等^[44]^回顾了298个高代谢淋巴结，为了区分高代谢淋巴结的良恶性，他们提取了PET上高代谢淋巴结区域的影像组学特征，并筛选出4个特征用于建立诊断模型，在外部验证队列中其AUC为0.808，而通过2个临床特征（淋巴结大小和淋巴结平均CT值）建立模型的AUC为0.802，结合三者的综合模型AUC为0.841，此外他们发现恶性淋巴结有3个与组织异质性相关的影像组学特征GLRLM_GLNU、GLRLM_RLNU及NGLDM_Coarseness显著高于良性淋巴结，这提示恶性淋巴结可能较良性淋巴结具有更高的组织异质性，这或许是影像组学诊断淋巴结良恶性的生物学基础之一。

通过影像组学提取并筛选淋巴结区域丰富的影像特征，进而建立诊断模型已提升了淋巴结良恶性诊断的准确性，表现出了巨大的临床价值，但目前研究以单中心回顾性研究为主，还有待进一步多中心前瞻性研究验证。

## 6 基于深度学习诊断肿大淋巴结良恶性

深度学习是一类先进的人工智能方法，它具有的多层神经网络架构可将输入信息转换为多个抽象层次的数据表示，极大地提升了图像、语音、文本等识别的水平^[45]^。对于医学影像处理，深度卷积神经网络（deep convolutional neural networks, DCNN）是最常用的深度学习网络，它可通过反向传播调整权重进行训练，从训练样本中学习提取相关特征^[46]^。相较于影像组学所提取的特征是预先设计的，DCNN通过训练来自动学习目标相关的特征表征，可以学习到更抽象和丰富的特征。此外相较于影像组学，深度学习无需精确地描绘ROI，大大减少了工作量。

目前在NSCLC中基于深度学习建立淋巴结良恶性诊断模型尚处于起步阶段，Pham等^[47]^分析了271个纵隔淋巴结的CT影像，通过预训练的深度学习网络ResNet-101提取影像特征，并通过支持向量机建立诊断模型，结果显示模型的准确性为88%，敏感性为85%，特异性为92%，较使用传统影像组学模型效果更佳。然而相较于传统的影像组学特征，深度学习网络提取的特征更加抽象，临床医生可能对其诊断结果持怀疑态度，但不可否认深度学习模型已在多种医学任务中表现出巨大优势^[48]^。在其他类型的肿瘤中，基于深度学习诊断淋巴结良恶性已取得了良好的效果，如Lee等^[49]^在甲状腺癌中应用995个颈部淋巴结的CT影像训练了基于深度学习的淋巴结良恶性诊断模型，其AUC为0.953，并后续进行了外部验证，其AUC为0.884^[50]^。

基于深度学习建立NSCLC淋巴结良恶性诊断模型，表现出了巨大的潜力，未来通过建立更先进的“端到端”人工智能模型，或许将进一步提升模型的诊断性能，同时相较于影像组学，可进一步减轻人工描画ROI的负担和主观性。

## 7 问题与展望

诊断NSCLC患者肿大淋巴结的良恶性是准确评估患者淋巴结分期的基础，但目前广泛使用的CT和PET/CT诊断标准假阳性率和假阴性率均较高，无法满足临床需求。CT诊断基于肿瘤细胞引起淋巴结结构（大小、密度等）改变，其敏感性和特异性均低。PET/CT诊断基于肿瘤细胞引起淋巴结代谢增高，敏感性明显高于CT，但炎症等同样可引起淋巴结代谢增高，因而特异性较低，DTP PET/CT的应用有助于进一步提升诊断性能。MRI诊断基于肿瘤细胞引起水分子运动受限，在良性肿大中较少出现，因而具有更高的特异性。目前影像学基于淋巴结结构或代谢改变进行诊断，需肿瘤细胞达一定数量，因而对微小转移灶的诊断仍存在困难，导致敏感性较低。影像组学在目前的成像技术下，通过挖掘丰富的影像特征并建立诊断模型，进一步提升了诊断准确性，表现出巨大的应用价值，但目前的研究主要以单中心回顾性研究为主，还有待进一步临床检验。基于单一模态影像已进行了大量研究，进而联合多模态影像数据以提升诊断准确性将是未来的研究重点，此外应用更先进的人工智能技术能否进一步提升诊断准确性也值得期待。
